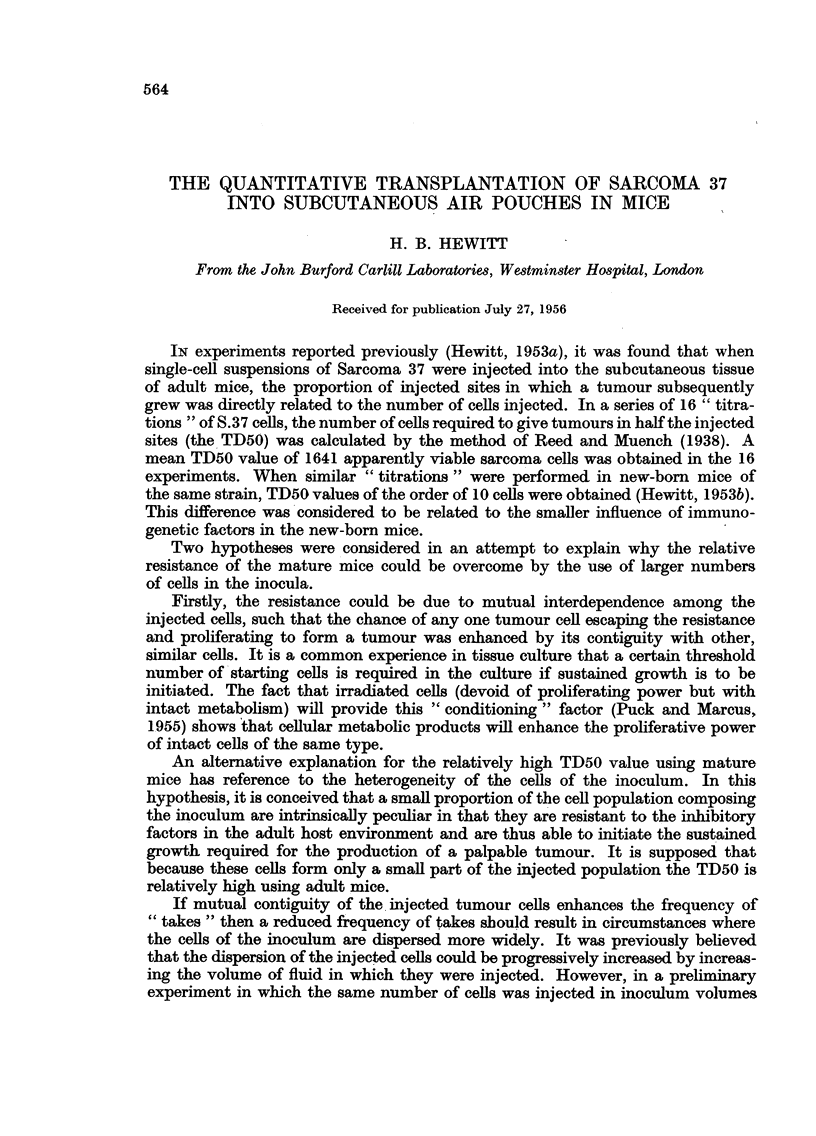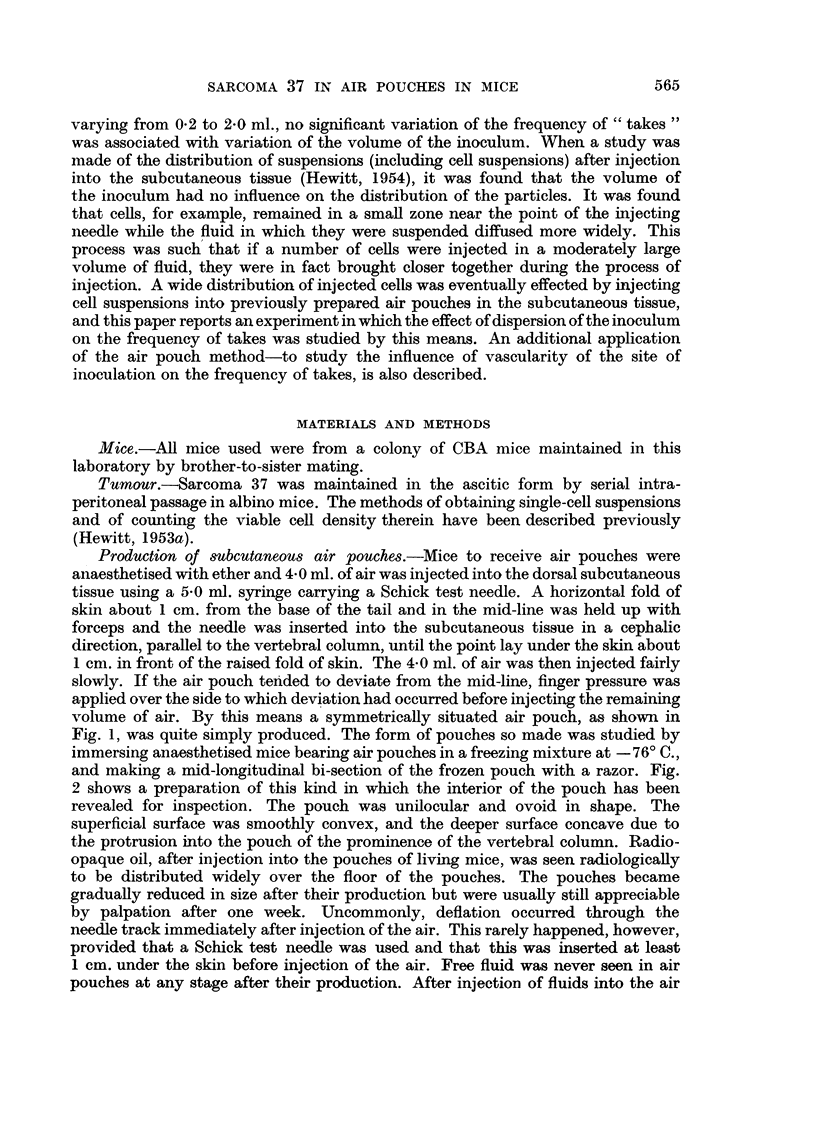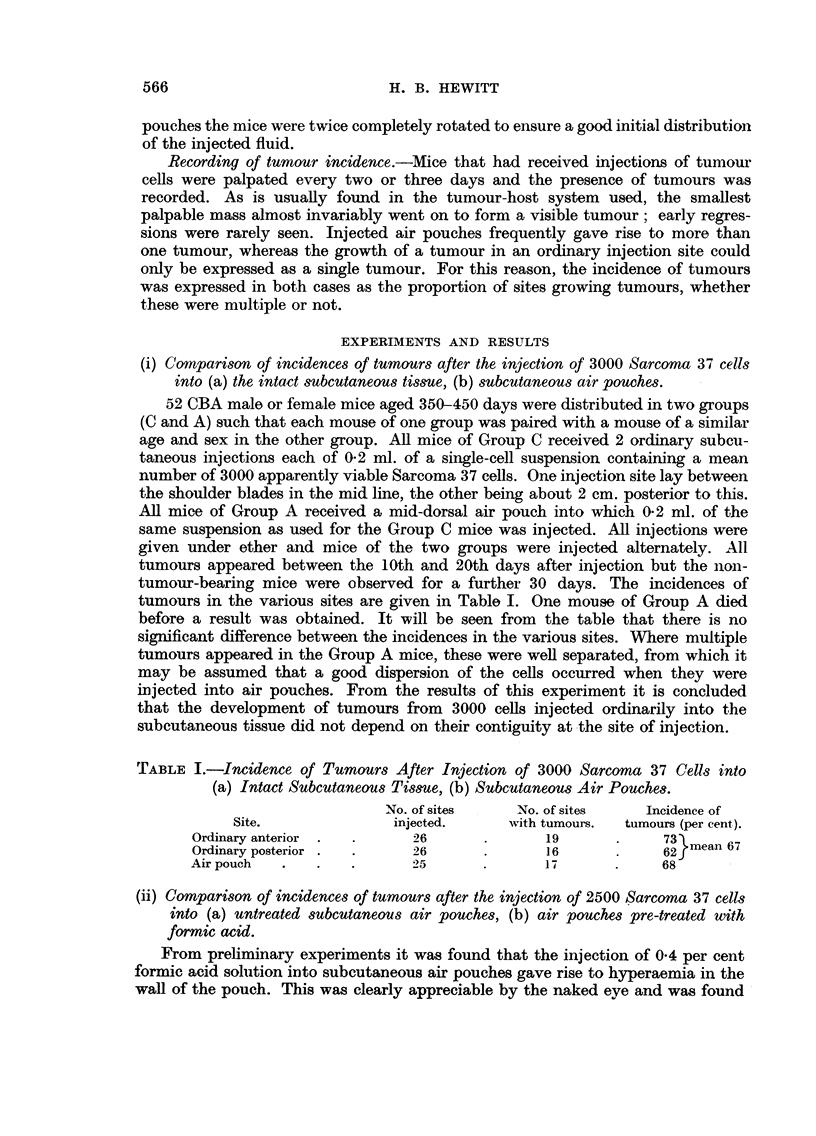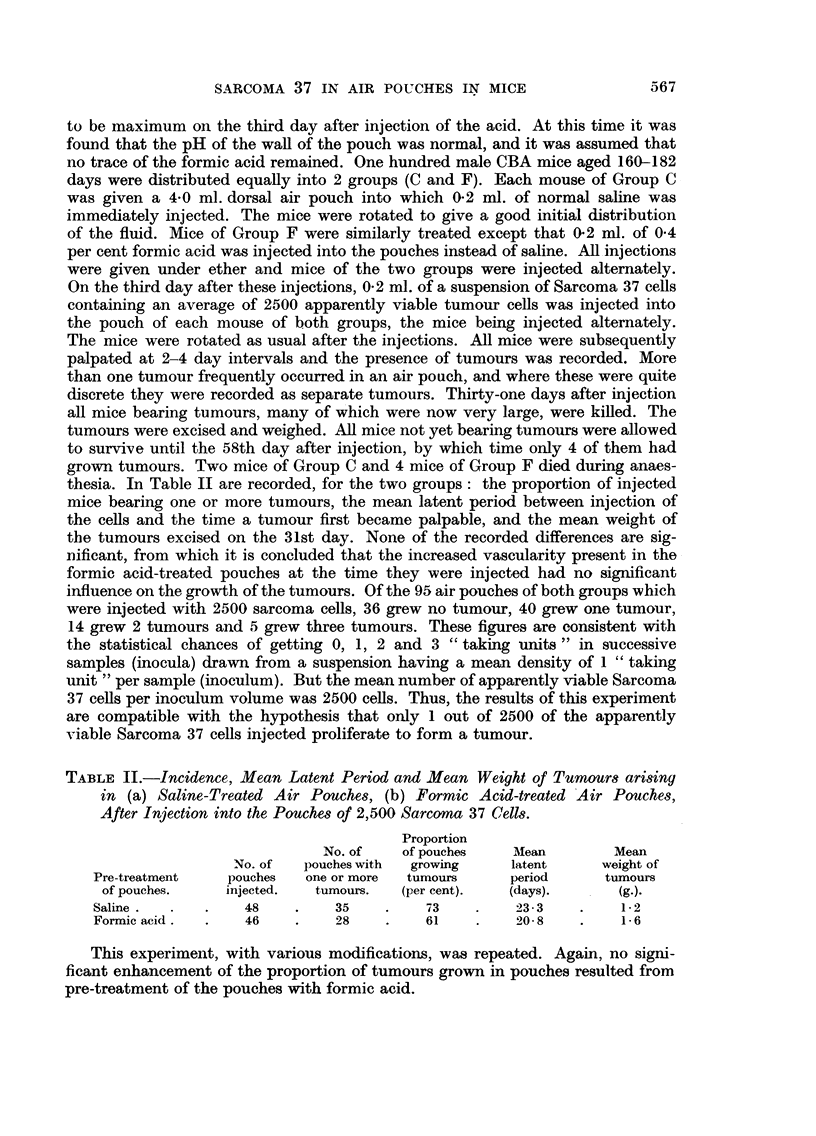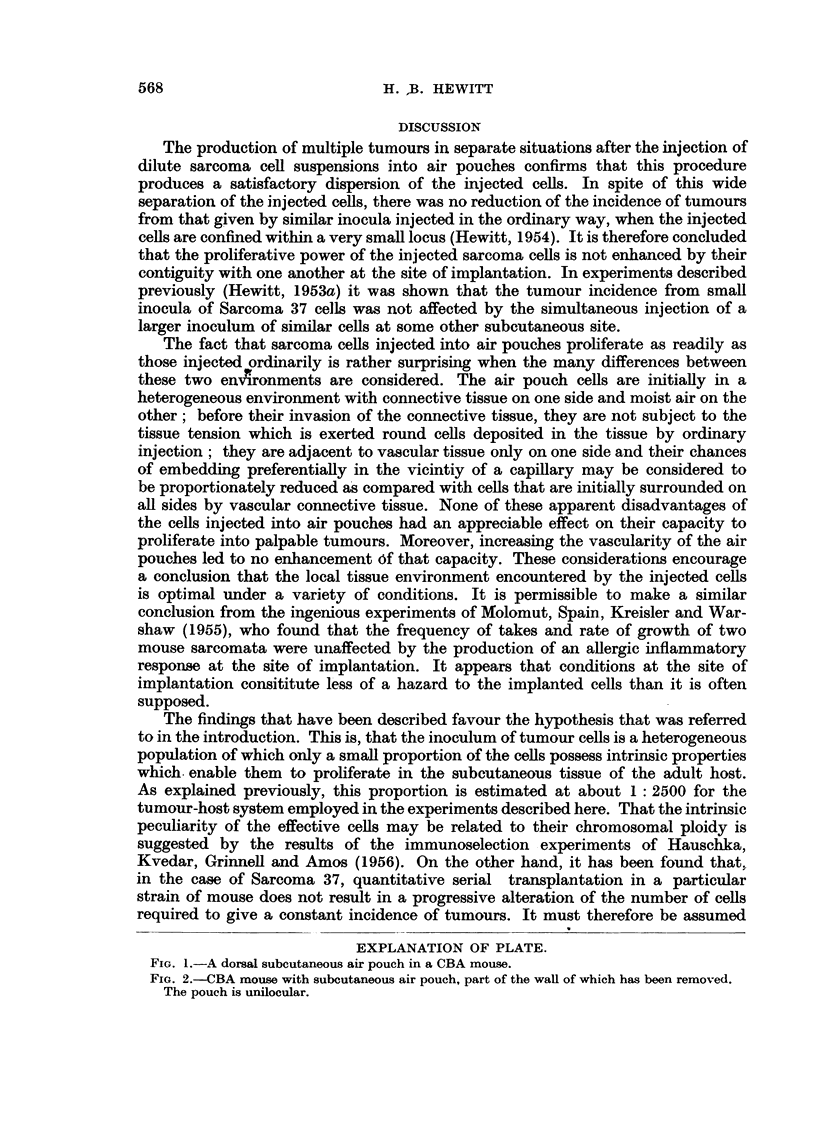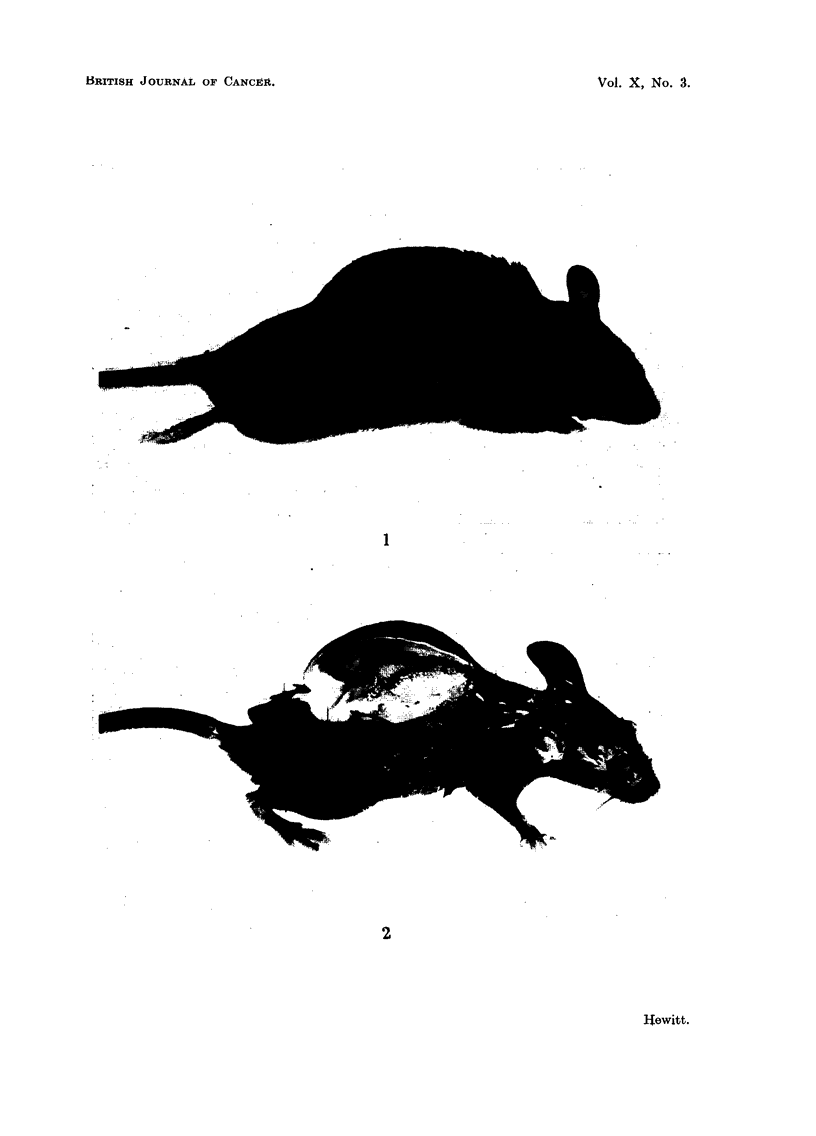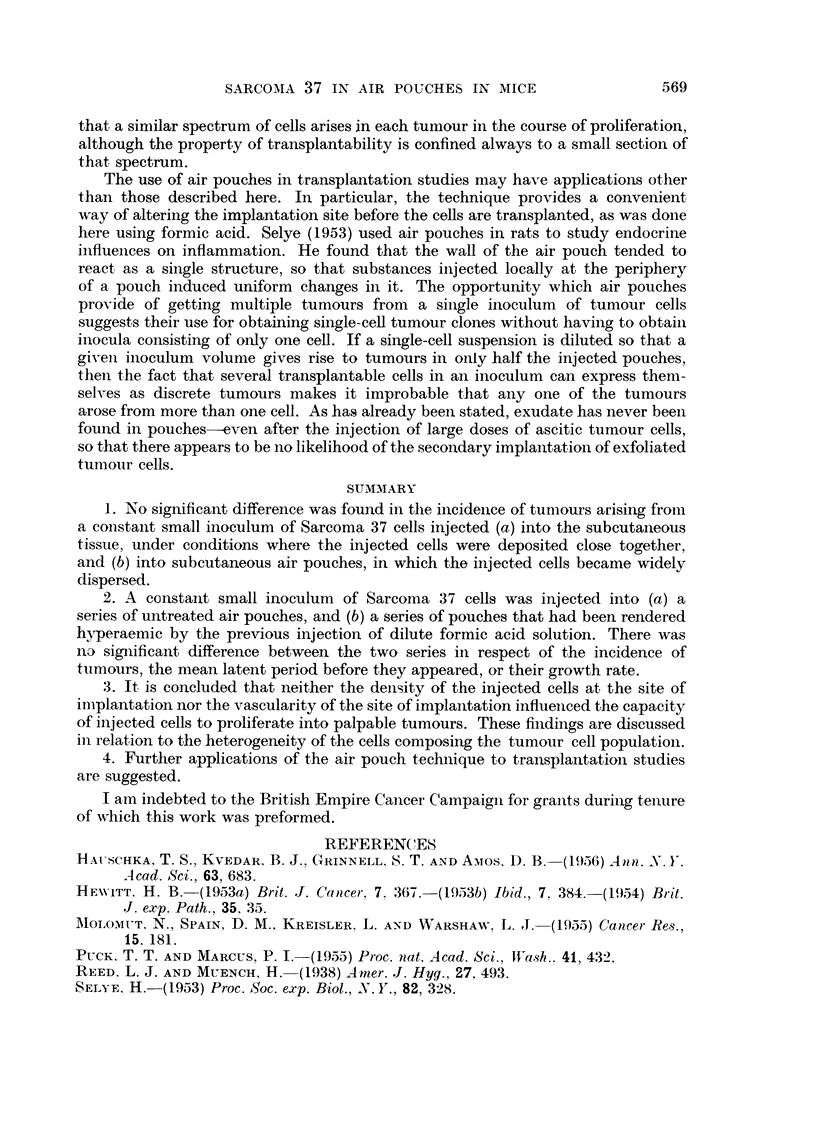# The Quantitative Transplantation of Sarcoma 37 into Subcutaneous Air Pouches in Mice

**DOI:** 10.1038/bjc.1956.67

**Published:** 1956-09

**Authors:** H. B. Hewitt

## Abstract

**Images:**


### THE QUANTITATIVE TRANSPLANTATION OF SARCOMA 37 INTO SUBCUTANEOUS AIR POUCHES IN MICE

H. B. HEWITT

*From the John Burford Carlill Laboratories, Westminster Hospital, London*

Received for publication July 27, 1956

In experiments reported previously (Hewitt, 1953*a*), it was found that when single-cell suspensions of Sarcoma 37 were injected into the subcutaneous tissue of adult mice, the proportion of injected sites in which a tumour subsequently grew was directly related to the number of cells injected. In a series of 16 " titrations " of S.37 cells, the number of cells required to give tumours in half the injected sites (the TD50) was calculated by the method of Reed and Muench (1938). A mean TD50 value of 1641 apparently viable sarcoma cells was obtained in the 16 experiments. When similar " titrations " were performed in new-born mice of the same strain, TD50 values of the order of 10 cells were obtained (Hewitt, 1953*b*). This difference was considered to be related to the smaller influence of immunogenetic factors in the new-born mice.

Two hypotheses were considered in an attempt to explain why the relative resistance of the mature mice could be overcome by the use of larger numbers of cells in the inocula.

Firstly, the resistance could be due to mutual interdependence among the injected cells, such that the chance of any one tumour cell escaping the resistance and proliferating to form a tumour was enhanced by its contiguity with other, similar cells. It is a common experience in tissue culture that a certain threshold number of starting cells is required in the culture if sustained growth is to be initiated. The fact that irradiated cells (devoid of proliferating power but with intact metabolism) will provide this " conditioning " factor (Puck and Marcus, 1955) shows that cellular metabolic products will enhance the proliferative power of intact cells of the same type.

An alternative explanation for the relatively high TD50 value using mature mice has reference to the heterogeneity of the cells of the inoculum. In this hypothesis, it is conceived that a small proportion of the cell population composing the inoculum are intrinsically peculiar in that they are resistant to the inhibitory factors in the adult host environment and are thus able to initiate the sustained growth required for the production of a palpable tumour. It is supposed that because these cells form only a small part of the injected population the TD50 is relatively high using adult mice.

If mutual contiguity of the injected tumour cells enhances the frequency of " takes " then a reduced frequency of takes should result in circumstances where the cells of the inoculum are dispersed more widely. It was previously believed that the dispersion of the injected cells could be progressively increased by increasing the volume of fluid in which they were injected. However, in a preliminary experiment in which the same number of cells was injected in inoculum volumes varying from 0·2 to 2·0 ml., no significant variation of the frequency of " takes " was associated with variation of the volume of the inoculum. When a study was made of the distribution of suspensions (including cell suspensions) after injection into the subcutaneous tissue (Hewitt, 1954), it was found that the volume of the inoculum had no influence on the distribution of the particles. It was found that cells, for example, remained in a small zone near the point of the injecting needle while the fluid in which they were suspended diffused more widely. This process was such that if a number of cells were injected in a moderately large volume of fluid, they were in fact brought closer together during the process of injection. A wide distribution of injected cells was eventually effected by injecting cell suspensions into previously prepared air pouches in the subcutaneous tissue, and this paper reports an experiment in which the effect of dispersion of the inoculum on the frequency of takes was studied by this means. An additional application of the air pouch method—to study the influence of vascularity of the site of inoculation on the frequency of takes, is also described.

### MATERIALS AND METHODS

*Mice.*—All mice used were from a colony of CBA mice maintained in this laboratory by brother-to-sister mating.

*Tumour.*—Sarcoma 37 was maintained in the ascitic form by serial intraperitoneal passage in albino mice. The methods of obtaining single-cell suspensions and of counting the viable cell density therein have been described previously (Hewitt, 1953*a*).

*Production of subcutaneous air pouches.*—Mice to receive air pouches were anaesthetised with ether and 4·0 ml. of air was injected into the dorsal subcutaneous tissue using a 5·0 ml. syringe carrying a Schick test needle. A horizontal fold of skin about 1 cm. from the base of the tail and in the mid-line was held up with forceps and the needle was inserted into the subcutaneous tissue in a cephalic direction, parallel to the vertebral column, until the point lay under the skin about 1 cm. in front of the raised fold of skin. The 4·0 ml. of air was then injected fairly slowly. If the air pouch tended to deviate from the mid-line, finger pressure was applied over the side to which deviation had occurred before injecting the remaining volume of air. By this means a symmetrically situated air pouch, as shown in Fig. 1, was quite simply produced. The form of pouches so made was studied by immersing anaesthetised mice bearing air pouches in a freezing mixture at —76° C., and making a mid-longitudinal bi-section of the frozen pouch with a razor. Fig. 2 shows a preparation of this kind in which the interior of the pouch has been revealed for inspection. The pouch was unilocular and ovoid in shape. The superficial surface was smoothly convex, and the deeper surface concave due to the protrusion into the pouch of the prominence of the vertebral column. Radio-opaque oil, after injection into the pouches of living mice, was seen radiologically to be distributed widely over the floor of the pouches. The pouches became gradually reduced in size after their production but were usually still appreciable by palpation after one week. Uncommonly, deflation occurred through the needle track immediately after injection of the air. This rarely happened, however, provided that a Schick test needle was used and that this was inserted at least 1 cm. under the skin before injection of the air. Free fluid was never seen in air pouches at any stage after their production. After injection of fluids into the air pouches the mice were twice completely rotated to ensure a good initial distribution of the injected fluid.

*Recording of tumour incidence.*—Mice that had received injections of tumour cells were palpated every two or three days and the presence of tumours was recorded. As is usually found in the tumour-host system used, the smallest palpable mass almost invariably went on to form a visible tumour ; early regressions were rarely seen. Injected air pouches frequently gave rise to more than one tumour, whereas the growth of a tumour in an ordinary injection site could only be expressed as a single tumour. For this reason, the incidence of tumours was expressed in both cases as the proportion of sites growing tumours, whether these were multiple or not.

### EXPERIMENTS AND RESULTS

(i) *Comparison of incidences of tumours after the injection of* 3000 *Sarcoma* 37 *cells into* (a) *the intact subcutaneous tissue,* (b) *subcutaneous air pouches.*

52 CBA male or female mice aged 350–450 days were distributed in two groups (C and A) such that each mouse of one group was paired with a mouse of a similar age and sex in the other group. All mice of Group C received 2 ordinary subcutaneous injections each of 0·2 ml. of a single-cell suspension containing a mean number of 3000 apparently viable Sarcoma 37 cells. One injection site lay between the shoulder blades in the mid line, the other being about 2 cm. posterior to this. All mice of Group A received a mid-dorsal air pouch into which 0·2 ml. of the same suspension as used for the Group C mice was injected. All injections were given under ether and mice of the two groups were injected alternately. All tumours appeared between the 10th and 20th days after injection but the non-tumour-bearing mice were observed for a further 30 days. The incidences of tumours in the various sites are given in Table I. One mouse of Group A died before a result was obtained. It will be seen from the table that there is no significant difference between the incidences in the various sites. Where multiple tumours appeared in the Group A mice, these were well separated, from which it may be assumed that a good dispersion of the cells occurred when they were injected into air pouches. From the results of this experiment it is concluded that the development of tumours from 3000 cells injected ordinarily into the subcutaneous tissue did not depend on their contiguity at the site of injection.

TABLE I.—*Incidence of Tumours After Injection of* 3000 *Sarcoma* 37 *Cells into* (a) *Intact Subcutaneous Tissue,* (b) *Subcutaneous Air Pouches.*

| Site. | No. of sites injected. | No. of sites with tumours. | Incidence of tumours (per cent). |
|---|---|---|---|
| Ordinary anterior | 26 | 19 | 73 } mean 67 |
| Ordinary posterior | 26 | 16 | 62 } mean 67 |
| Air pouch | 25 | 17 | 68 |

(ii) *Comparison of incidences of tumours after the injection of* 2500 *Sarcoma* 37 *cells into* (a) *untreated subcutaneous air pouches,* (b) *air pouches pre-treated with formic acid.*

From preliminary experiments it was found that the injection of 0·4 per cent formic acid solution into subcutaneous air pouches gave rise to hyperaemia in the wall of the pouch. This was clearly appreciable by the naked eye and was found to be maximum on the third day after injection of the acid. At this time it was found that the pH of the wall of the pouch was normal, and it was assumed that no trace of the formic acid remained. One hundred male CBA mice aged 160–182 days were distributed equally into 2 groups (C and F). Each mouse of Group C was given a 4·0 ml. dorsal air pouch into which 0·2 ml. of normal saline was immediately injected. The mice were rotated to give a good initial distribution of the fluid. Mice of Group F were similarly treated except that 0·2 ml. of 0·4 per cent formic acid was injected into the pouches instead of saline. All injections were given under ether and mice of the two groups were injected alternately. On the third day after these injections, 0·2 ml. of a suspension of Sarcoma 37 cells containing an average of 2500 apparently viable tumour cells was injected into the pouch of each mouse of both groups, the mice being injected alternately. The mice were rotated as usual after the injections. All mice were subsequently palpated at 2–4 day intervals and the presence of tumours was recorded. More than one tumour frequently occurred in an air pouch, and where these were quite discrete they were recorded as separate tumours. Thirty-one days after injection all mice bearing tumours, many of which were now very large, were killed. The tumours were excised and weighed. All mice not yet bearing tumours were allowed to survive until the 58th day after injection, by which time only 4 of them had grown tumours. Two mice of Group C and 4 mice of Group F died during anaesthesia. In Table II are recorded, for the two groups : the proportion of injected mice bearing one or more tumours, the mean latent period between injection of the cells and the time a tumour first became palpable, and the mean weight of the tumours excised on the 31st day. None of the recorded differences are significant, from which it is concluded that the increased vascularity present in the formic acid-treated pouches at the time they were injected had no significant influence on the growth of the tumours. Of the 95 air pouches of both groups which were injected with 2500 sarcoma cells, 36 grew no tumour, 40 grew one tumour, 14 grew 2 tumours and 5 grew three tumours. These figures are consistent with the statistical chances of getting 0, 1, 2 and 3 " taking units " in successive samples (inocula) drawn from a suspension having a mean density of 1 " taking unit " per sample (inoculum). But the mean number of apparently viable Sarcoma 37 cells per inoculum volume was 2500 cells. Thus, the results of this experiment are compatible with the hypothesis that only 1 out of 2500 of the apparently viable Sarcoma 37 cells injected proliferate to form a tumour.

TABLE II.—*Incidence, Mean Latent Period and Mean Weight of Tumours arising in* (a) *Saline-Treated Air Pouches,* (b) *Formic Acid-treated Air Pouches, After Injection into the Pouches of* 2,500 *Sarcoma* 37 *Cells.*

| Pre-treatment of pouches. | No. of pouches injected. | No. of pouches with one or more tumours. | Proportion of pouches growing tumours (per cent). | Mean latent period (days). | Mean weight of tumours (g.). |
|---|---|---|---|---|---|
| Saline | 48 | 35 | 73 | 23·3 | 1·2 |
| Formic acid | 46 | 28 | 61 | 20·8 | 1·6 |

This experiment, with various modifications, was repeated. Again, no significant enhancement of the proportion of tumours grown in pouches resulted from pre-treatment of the pouches with formic acid.

### DISCUSSION

The production of multiple tumours in separate situations after the injection of dilute sarcoma cell suspensions into air pouches confirms that this procedure produces a satisfactory dispersion of the injected cells. In spite of this wide separation of the injected cells, there was no reduction of the incidence of tumours from that given by similar inocula injected in the ordinary way, when the injected cells are confined within a very small locus (Hewitt, 1954). It is therefore concluded that the proliferative power of the injected sarcoma cells is not enhanced by their contiguity with one another at the site of implantation. In experiments described previously (Hewitt, 1953*a*) it was shown that the tumour incidence from small inocula of Sarcoma 37 cells was not affected by the simultaneous injection of a larger inoculum of similar cells at some other subcutaneous site.

The fact that sarcoma cells injected into air pouches proliferate as readily as those injected ordinarily is rather surprising when the many differences between these two environments are considered. The air pouch cells are initially in a heterogeneous environment with connective tissue on one side and moist air on the other ; before their invasion of the connective tissue, they are not subject to the tissue tension which is exerted round cells deposited in the tissue by ordinary injection ; they are adjacent to vascular tissue only on one side and their chances of embedding preferentially in the vicintiy of a capillary may be considered to be proportionately reduced as compared with cells that are initially surrounded on all sides by vascular connective tissue. None of these apparent disadvantages of the cells injected into air pouches had an appreciable effect on their capacity to proliferate into palpable tumours. Moreover, increasing the vascularity of the air pouches led to no enhancement of that capacity. These considerations encourage a conclusion that the local tissue environment encountered by the injected cells is optimal under a variety of conditions. It is permissible to make a similar conclusion from the ingenious experiments of Molomut, Spain, Kreisler and Warshaw (1955), who found that the frequency of takes and rate of growth of two mouse sarcomata were unaffected by the production of an allergic inflammatory response at the site of implantation. It appears that conditions at the site of implantation consititute less of a hazard to the implanted cells than it is often supposed.

The findings that have been described favour the hypothesis that was referred to in the introduction. This is, that the inoculum of tumour cells is a heterogeneous population of which only a small proportion of the cells possess intrinsic properties which enable them to proliferate in the subcutaneous tissue of the adult host. As explained previously, this proportion is estimated at about 1 : 2500 for the tumour-host system employed in the experiments described here. That the intrinsic peculiarity of the effective cells may be related to their chromosomal ploidy is suggested by the results of the immunoselection experiments of Hauschka, Kvedar, Grinnell and Amos (1956). On the other hand, it has been found that, in the case of Sarcoma 37, quantitative serial transplantation in a particular strain of mouse does not result in a progressive alteration of the number of cells required to give a constant incidence of tumours. It must therefore be assumed

---

EXPLANATION OF PLATE.

FIG. 1.—A dorsal subcutaneous air pouch in a CBA mouse.

FIG. 2.—CBA mouse with subcutaneous air pouch, part of the wall of which has been removed. The pouch is unilocular.

1

2

Hewitt.

that a similar spectrum of cells arises in each tumour in the course of proliferation, although the property of transplantability is confined always to a small section of that spectrum.

The use of air pouches in transplantation studies may have applications other than those described here. In particular, the technique provides a convenient way of altering the implantation site before the cells are transplanted, as was done here using formic acid. Selye (1953) used air pouches in rats to study endocrine influences on inflammation. He found that the wall of the air pouch tended to react as a single structure, so that substances injected locally at the periphery of a pouch induced uniform changes in it. The opportunity which air pouches provide of getting multiple tumours from a single inoculum of tumour cells suggests their use for obtaining single-cell tumour clones without having to obtain inocula consisting of only one cell. If a single-cell suspension is diluted so that a given inoculum volume gives rise to tumours in only half the injected pouches, then the fact that several transplantable cells in an inoculum can express themselves as discrete tumours makes it improbable that any one of the tumours arose from more than one cell. As has already been stated, exudate has never been found in pouches—even after the injection of large doses of ascitic tumour cells, so that there appears to be no likelihood of the secondary implantation of exfoliated tumour cells.

### SUMMARY

1. No significant difference was found in the incidence of tumours arising from a constant small inoculum of Sarcoma 37 cells injected (*a*) into the subcutaneous tissue, under conditions where the injected cells were deposited close together, and (*b*) into subcutaneous air pouches, in which the injected cells became widely dispersed.

2. A constant small inoculum of Sarcoma 37 cells was injected into (*a*) a series of untreated air pouches, and (*b*) a series of pouches that had been rendered hyperaemic by the previous injection of dilute formic acid solution. There was no significant difference between the two series in respect of the incidence of tumours, the mean latent period before they appeared, or their growth rate.

3. It is concluded that neither the density of the injected cells at the site of implantation nor the vascularity of the site of implantation influenced the capacity of injected cells to proliferate into palpable tumours. These findings are discussed in relation to the heterogeneity of the cells composing the tumour cell population.

4. Further applications of the air pouch technique to transplantation studies are suggested.

I am indebted to the British Empire Cancer Campaign for grants during tenure of which this work was preformed.

### REFERENCES

HAUSCHKA, T. S., KVEDAR, B. J., GRINNELL, S. T. AND AMOS, D. B.—(1956) *Ann. N.Y. Acad. Sci.*, **63**, 683.

HEWITT, H. B.—(1953*a*) *Brit. J. Cancer*, **7**, 367.—(1953*b*) *Ibid.*, **7**, 384.—(1954) *Brit. J. exp. Path.*, **35**, 35.

MOLOMUT, N., SPAIN, D. M., KREISLER, L. AND WARSHAW, L. J.—(1955) *Cancer Res.*, **15**, 181.

PUCK, T. T. AND MARCUS, P. I.—(1955) *Proc. nat. Acad. Sci., Wash.*, **41**, 432.

REED, L. J. AND MUENCH, H.—(1938) *Amer. J. Hyg.*, **27**, 493.

SELYE, H.—(1953) *Proc. Soc. exp. Biol., N.Y.*, **82**, 328.